# *GhAAO2* was observed responding to NaHCO_3_ stress in cotton compared to *AAO* family genes

**DOI:** 10.1186/s12870-022-03999-7

**Published:** 2022-12-20

**Authors:** Xiaoyu Liu, Yupeng Cui, Ruiqin Kang, Hong Zhang, Hui Huang, Yuqian Lei, Yapeng Fan, Yuexin Zhang, Jing Wang, Nan Xu, Mingge Han, Xixian Feng, Kesong Ni, Tiantian Jiang, Cun Rui, Liangqing Sun, Xiugui Chen, Xuke Lu, Delong Wang, Junjuan Wang, Shuai Wang, Lanjie Zhao, Lixue Guo, Chao Chen, Quanjia Chen, Wuwei Ye

**Affiliations:** 1 Institute of Cotton Research of Chinese Academy of Agricultural Sciences / Zhengzhou Research Base, State Key Laboratory of Cotton Biology, School of Agricultural Sciences, Zhengzhou University, Anyang, 455000 Henan China; 2 Engineering Research Centre of Cotton, Ministry of Education / College of Agriculture, Xinjiang Agricultural University, 311 Nongda East Road, Urumqi, 830052 China; 3 Anyang Institute of Technology, Anyang, 455000 Henan China

**Keywords:** Abscisic acid aldehyde oxidase (AAO), *Gossypium hirsutum*, Abiotic stresses, Gene family, Functional Verification

## Abstract

**Background:**

Abscisic acid (ABA) is an important stress hormone, the changes of abscisic acid content can alter plant tolerance to stress, abscisic acid is crucial for studying plant responses to abiotic stress. The abscisic acid aldehyde oxidase (AAO) plays a vital role in the final step in the synthesis of abscisic acid, therefore, understanding the function of *AAO* gene family is of great significance for plants to response to abiotic stresses.

**Result:**

In this study, 6, 8, 4 and 4 *AAO* genes were identified in four cotton species. According to the structural characteristics of genes and the traits of phylogenetic tree, we divided the *AAO* gene family into 4 clades. Gene structure analysis showed that the *AAO* gene family was relatively conservative. The analysis of *cis*-elements showed that most *AAO* genes contained *cis*-elements related to light response and plant hormones. Tissue specificity analysis under NaHCO_3_ stress showed that *GhAAO2* gene was differentially expressed in both roots and leaves. After *GhAAO2* gene silencing, the degree of wilting of seedlings was lighter than that of the control group, indicating that *GhAAO2* could respond to NaHCO_3_ stress.

**Conclusions:**

In this study, the *AAO* gene family was analyzed by bioinformatics, the response of *GhAAO* gene to various abiotic stresses was preliminarily verified, and the function of the specifically expressed gene *GhAAO2* was further verified. These findings provide valuable information for the study of potential candidate genes related to plant growth and stress.

**Supplementary Information:**

The online version contains supplementary material available at 10.1186/s12870-022-03999-7

## Background

Various biotic and abiotic stresses seriously affect cotton yield and fiber quality during its growth. To cope with the adverse environment, various signaling pathways that plants depend on have evolved many adaptive mechanisms [1]. Signaling pathways regulated by various plant hormones play an important role in defense against abiotic stresses, including Abscisic acid (ABA) [2], Salicylic acid (SA) [3], Jasmonic acid (JA) [4], and Ethylene (ET) [5]. Abscisic acid belongs to isoprene (terpenoids) [6], which plays an important role in promoting plant senescence, inhibiting plant growth, regulating embryonic development, seed dormancy, root development and fruit ripening [7]. It is an important secondary metabolite and stress hormone, which plays a vital role in abiotic stress. When plants are subjected to heat, salt, drought, cold and herbicides, the ABA levels in plants would be affected [8]. In the adversity environment, plants could synthesize abscisic acid, which can reduce the leaves expansion, induce the accumulation of proline, soluble sugar accumulation, promote the active oxygen metabolism, reduce the accumulation of malondialdehyde, regulate stomatal movement [9], which can strengthen the ability of plant resistance to adversity [10]. Abscisic acid has great potential application value in plant growth, development and coping with abiotic stress [11].

In recent years, the research on abscisic acid has made great progress. The biochemical, molecular genetics and pharmacology methods were used to determine the synthesis pathway of abscisic acid, the enzymes needed for the synthesis of ABA and the related genes catalyzing these enzymes were analyzed. It has been suggested that there may be two ABA biosynthesis pathways in higher plants: (1) the direct pathway is the polymerization of three isopentane units into the C15 precursor farnesyl pyrophosphate (FPP), which is composed of FPP through epoxidation and oxidation directly from 15-carbon ABA [12]; (2) In the indirect pathway, ABA is synthesized from carotenoids as precursors. Zeaxanthin epoxidase (ZEP) catalyzes cyclic zeaxanthin to form violaxanthin, which is cleaved to flavin aldehyde by 9-*cis*-epoxycarotenoid dioxygenase (NCED), Abscisic acid aldehyde is generated by short-chain dehydrogenase (SDR), finally, abscisic acid aldehyde is oxidized to ABA by abscisic acid aldehyde oxidase (AAO) [13]. There is now growing evidence that ABA is dominated by the C40 indirect biosynthetic pathway in higher plants, and it was confirmed that zeaxanthin epoxidase (ZEP), 9-*cis*-epoxycarotenoid dioxygenase (NCED), short-chain alcohol dehydrogenase / reductase (SDR) and ABA aldehyde oxidase (AAO) [12] are important enzymes in the ABA synthesis pathway. *AAO* gene was researched on the last step of ABA biosynthesis began in the 1970s [14], ABA aldehyde oxidase present in plants was first discovered in *Arabidopsis* [15]. This enzyme consist a multi-component protein containing a molybdenum cofactor, two iron sulfur cluster, and flavin adenine dinucleotide (FAD) as prosthetic gene, which is composed of a multicomponent protein [16].

*Arabidopsis* AOα and AOβ are homodimers of A01 and A02 products, respectively, A0γ is a heterodimer of these two gene products, AOδ is encoded by A03 gene and has the properties of ABA aldehyde oxidase [15]. AOδ both catalyzes the oxidation of ABA aldehyde to ABA and catalyzes the oxidation of 3-indole aldehyde to IAA. The Km value between two substrates is different. When ABA aldehyde is the substrate, the Km value is 0.51 umol / L, which is lower than that of 3-indole aldehyde oxidation. The Km value of the reaction showed that A0δ mainly acts on ABA biosynthesis. The oxidation product was confirmed by gas-mass spectrometry (GC–MS) to be ABA, this enzyme cannot distinguish between ABA aldehyde ( +) and (-) enantiomers [15]. AAO is derived from XDH replication during the evolutionary process. Aldehyde oxidase is similar to xanthine oxidase and bisulfite oxidase, which belongs to molybdenum-flavin protein family [17]. The amino acid sequences of AAO and XDH are highly similar, but their catalytic mechanisms are obviously different. Aldehyde oxidase does not participate in electron transfer in the form of dehydrogenase during the catalytic process [18]. However, the structure of sulfite oxidase is significantly different from the aldehyde oxidase [19]. Aldehyde oxidase can catalyze the oxidation and reduction of a variety of compounds, and its substrates include aldehydes, nitroso compounds, imines and heterocyclic compounds. AAO has a broader substrate specificity than XDH [20].

## Results

### Identification of *AAO* family members

We identified 6, 8, 4 and 4 *AAO* genes from the whole genome identification analysis of four cotton species (*G. hirsutum*, *G. barbadense*, *G. arboreum* and *G. raimondii*). A total of 22 *AAO* gene family members were identified, which renamed according to their chromosomal location distribution and *Arabidopsis* comparison results. The number of *AAO* genes in two tetraploid cotton cultivars, *G. hirsutum* and *G. barbadense*, was found to be almost twice that of the two diploid cotton cultivars *G. arboreum* and *G. raimondii*, which indicating that *AAO* genes have undergone amplified during evolution [21].

**Fig. 1:**
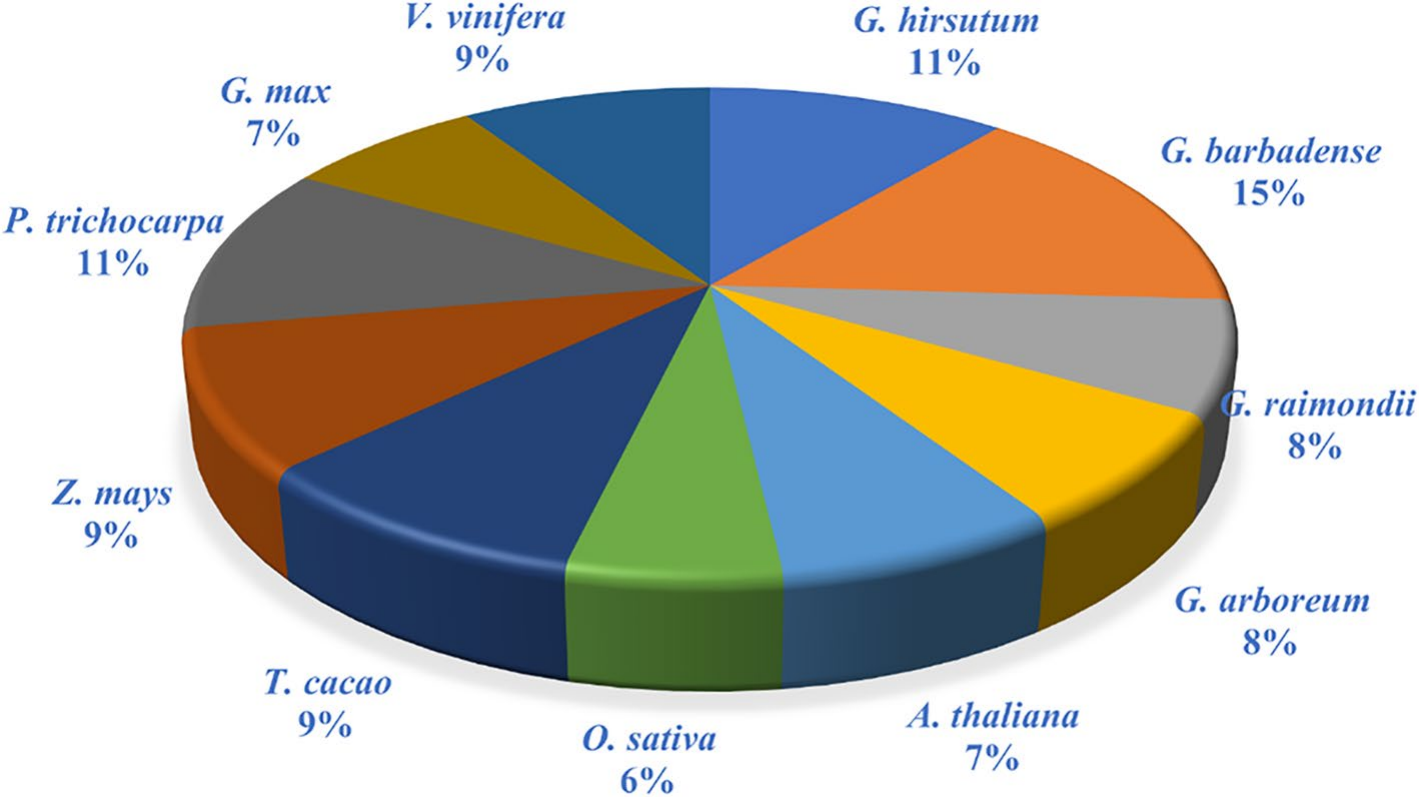
Distribution of *AAO* genes among eleven plant species

To clarify the evolutionary relationship between four cotton species and other closely related species, 7 species of *AAO* family members with close homology to cotton were identified by Blastp with evalue 1e-5, 4 from *Arabidopsis thaliana*, 5 from *Vitis vinifera*, 3 from *Oryza sativa*, 5 from *Zea mays*, 4 from *Glycine max*, 6 from *Populus trichocarpa*, and 5 from *Theobroma cacao* (Table S1, Fig. 1).

### Sequence and evolution analysis

**Fig. 2:**
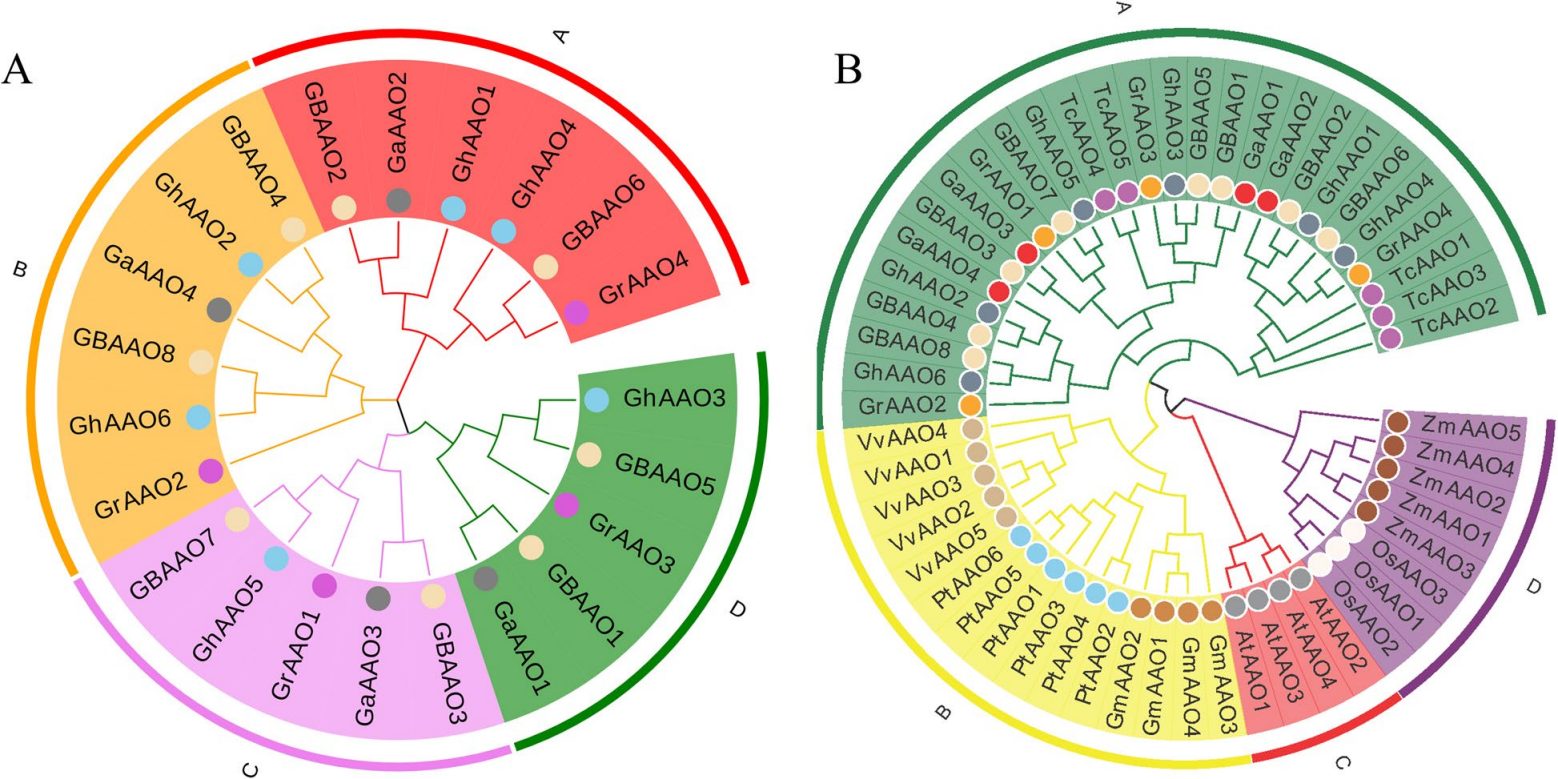
Two unrooted phylogenetic trees constructed by the Maximum Likelihood (ML) method of MEGA7. **A** A phylogenetic tree of 22 *AAO* genes in four cotton species. **B** Phylogenetic relationship of the 54 identified *AAO* genes from four cotton species and seven other plant species

To explore the evolutionary and orthologous relationship between four cotton species, 22 *AAO* genes were used for multiple sequence alignment. Subsequently, the phylogenetic tree constructed by using the Maximum Likelihood (ML) method and using the model as JTT + G with default parameters in MEGA software (Fig. 2A). To understand the evolutionary and orthologous relationship of 54 *AAO* protein sequences (22 from *Gossypium*, 4 from At, 3 from Os, 6 from Pt, 5 from Vv, 5 from Tc, 5 from Zm and 4 from Gm), we constructed the phylogenetic tree with JTT + G + I model using Maximum Likelihood (ML) method (Fig. 2B). The website EvolView (https://evolgenius.info//evolview-v2/#login) was used to beautify the obtained the two phylogenetic trees.

According to its branch on the evolutionary tree, we divided it into 4 subfamilies. From the evolutionary tree, we could find that *AAO* genes were relatively conserved in various species, and the *AAO* genes of the same species were all distributed on the same branch. *AAO* genes in cotton are closely related to *Theobroma cacao*, distributed on the same branch, *Vitis vinifera*, *Populus trichocarpa* and *Glycine max* were distributed on the same branch, *Oryza sativa* and *Zea mays* were distributed on the same branch, *Arabidopsis thaliana* was independent distribution.

### Chromosome distribution analysis

**Fig. 3:**
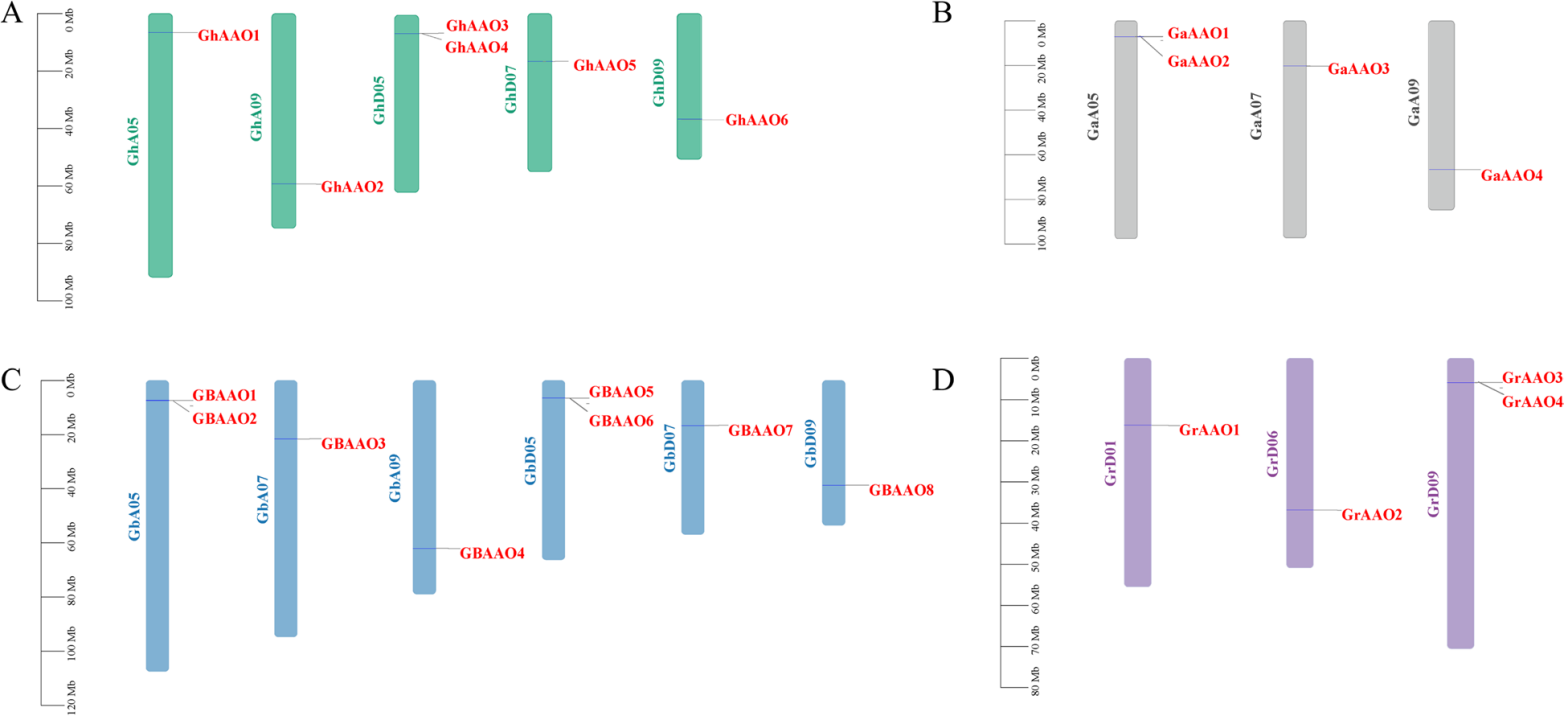
Chromosome distribution of *AAO* genes in four cotton species. Chromosomes of different cotton species are represented by different colors. The chromosomes of *G. hirsutum* are green. The chromosomes of *G. barbadense* are blue. The chromosomes of *G. arboreum* are gray. The chromosomes of *G. raimondii* are purple

The chromosome distribution of *AAO* genes family members help us understand the specific distribution more intuitively. The results showed that *AAO* gene family were mainly distributed on chromosomes 05, 07, 09 in *G. hirsutum* A and D (Fig. 3). There were 2 *AAO* genes on the A chromosome and 4 *AAO* genes on the D chromosome. In addition, the chromosomes distribution of genes of four *Gossypium* were not uniform. The *AAO* genes in *G. raimondii* were located on the 01, 06, and 09 in D chromosome, it is quite different from the other three cotton species, probably because *G. raimondii* is wild cotton, other three cotton species were cultivated cotton, which underwent artificial selection during evolution, the incomplete genome assembly or gene loss in the evolutionary process of the cotton species could be considered. All 22 *AAO* genes were mapped on chromosomes, indicating that *AAO* genes have matured in evolution.

### Analysis of conservative protein motif and gene structure

**Fig. 4:**
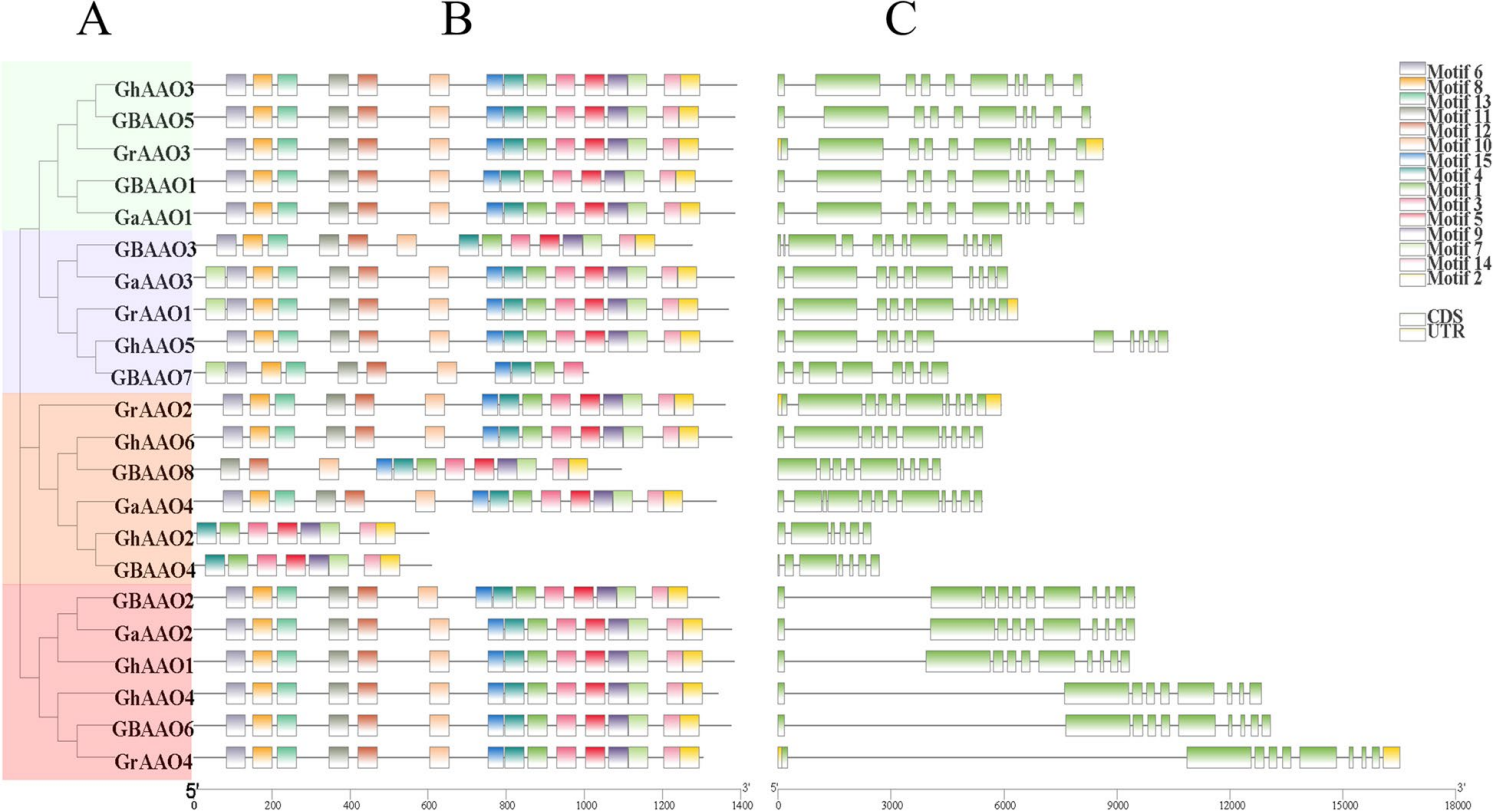
Conserved motifs and exon–intron structure analysis of *AAO* genes. **A** Phylogenetic tree of AAO proteins. **B** Conserved motifs of AAO proteins. **C** Exon–intron structures of *AAO* genes

To explore the conservative structure of *AAO* gene family, the conserved motif of the *AAO* genes were identified by the online website MEME, the evolutionary relationship, motif, coding, and non-coding regions of *AAO* gene family members were visualized in TBtools (Fig. 4). The *GBAAO8* gene lacked motif 6, motif 8 and motif 11, *GhAAO2* and *GBAAO4* lacked motif 6 motif 8, motif 11, motif 12, motif 10 and motif 15. The structure of the *AAO* genes are almost consistent, this is consistent with the finding that *AAO* is relatively conserved in the same species.

### Analysis of promoters of *AAO* gene family

**Fig. 5:**
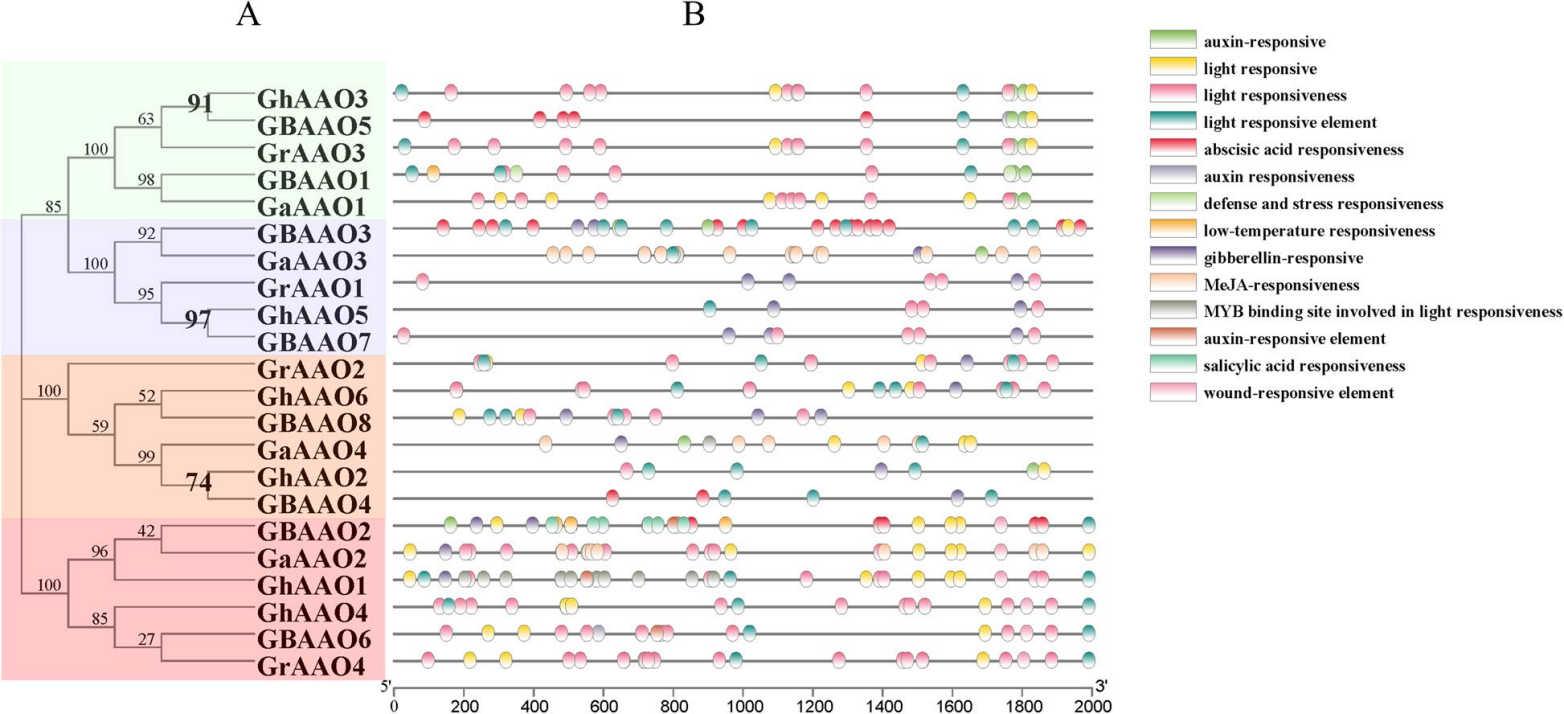
Analysis of promoters and differentially expressed *AAO* gene family members. **A** Phylogenetic tree of *AAO* gene family. **B** *Cis*-elements in promoters of *AAO* gene family members

Through website prediction and artificial selection, 17 *cis*-element related stress response element in the family of *AAO* were predicted (Fig. 5), which contain environmental stress, plant hormone and light response. Containing most *cis*-elements in the branch of *G. hirsutum GhAAO1* and *GhAAO4* and their close relatives, *cis*-acting elements are involved in the regulation of gene expression. We speculate that these genes may be sensitive to the external environment, when environmental have changes a greater may impact on these genes, the expression of these genes may easily have changed. Other genes in the *AAO* family also contain different *cis*-acting elements. They may be affected by light, plant hormones, abiotic stress and other conditions, and their expression levels may difference. Therefore, *AAO* genes may play an important role in regulating cotton growth and defending against the external environment.

### Gene duplication and collinearity analysis

**Fig. 6:**
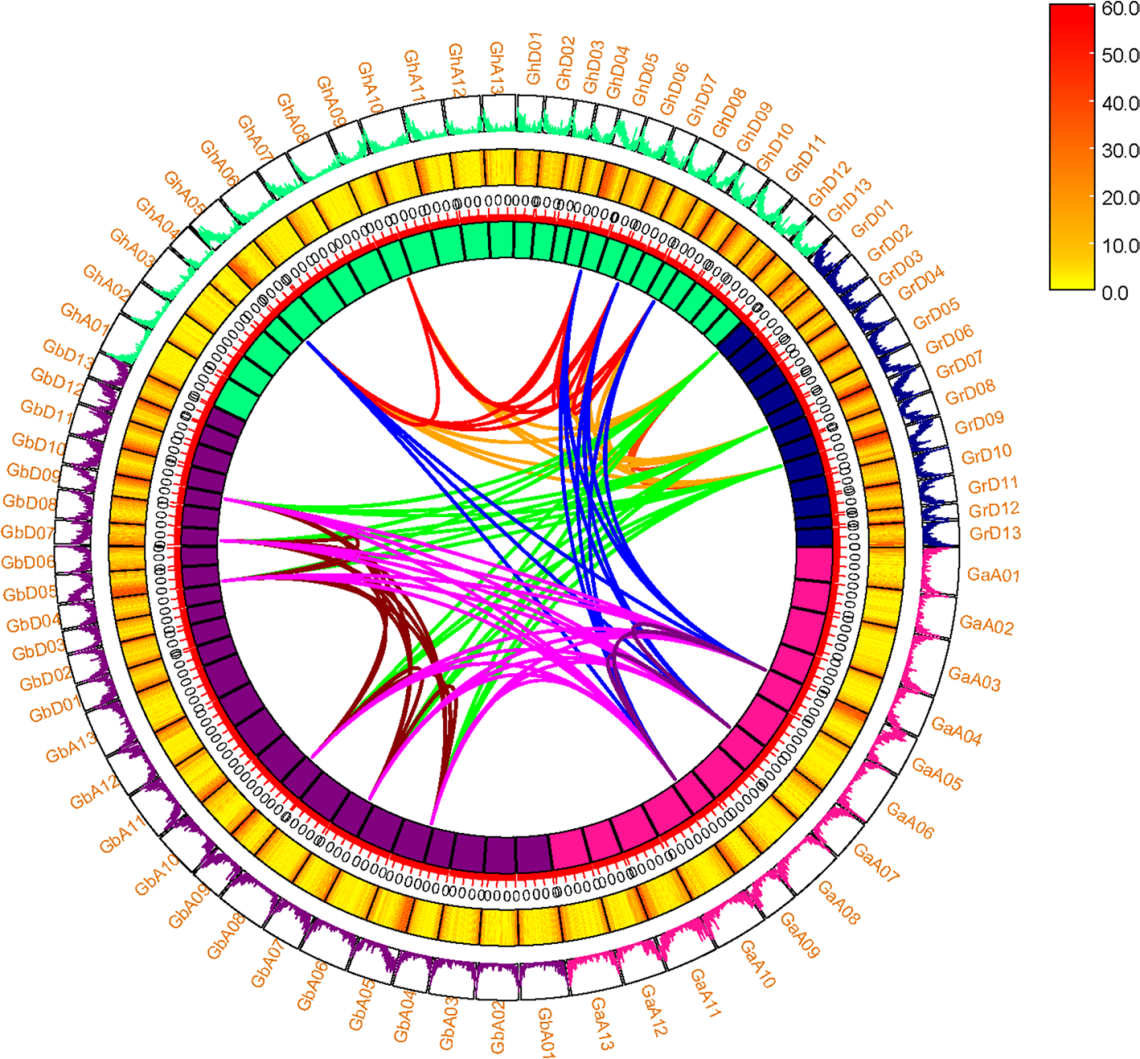
Syntenic relationship of duplicated gene pairs from four cotton species (*G. hirsutum*, *G. barbadense*, *G. arboreum* and *G. raimondii*). Chromosomal lines represented by various colors indicate the syntenic regions around the *AAO* genes. The heatmap and line map of the outer ring represents the density of genes on chromosomes

It was observed that two species with a highly conservative homology relationship have common linked genes from gene collinearity [22]. Gene duplication is one of the important reasons to promote the evolution of genome and genetic system [23]. The expansion of plant gene family mainly depends on whole genome duplication, fragment duplication and tandem duplication [24]. A total of 125 gene pairs were identified in 10 groups (Ga-Ga, Ga-GB, Ga-Gh, Ga-Gr, GB-GB, GB-Gh, GB-Gr, Gh-Gh, Gr-Gr), 31 homologous gene pairs were identified by self-comparison in Ga-Ga, GB-GB, Gh-Gh and Gr-Gr, which were predicted to be fragment duplication. 94 homologous gene pairs were identified by other combinations, which were predicted to be whole genome duplication. There were 25 pairs of GB-Gh homologous gene pairs were the most in 10 group, and 3 pairs of Gr-Gr and Ga-Ga homologous gene pairs were the least. It is speculated that fragment duplication and whole genome duplication were mainly carried out in the process of *AAO* genes evolution (Fig. 6).

### Analysis and calculation of selection pressure (*Ka* / *Ks*) during evolution

**Fig. 7:**
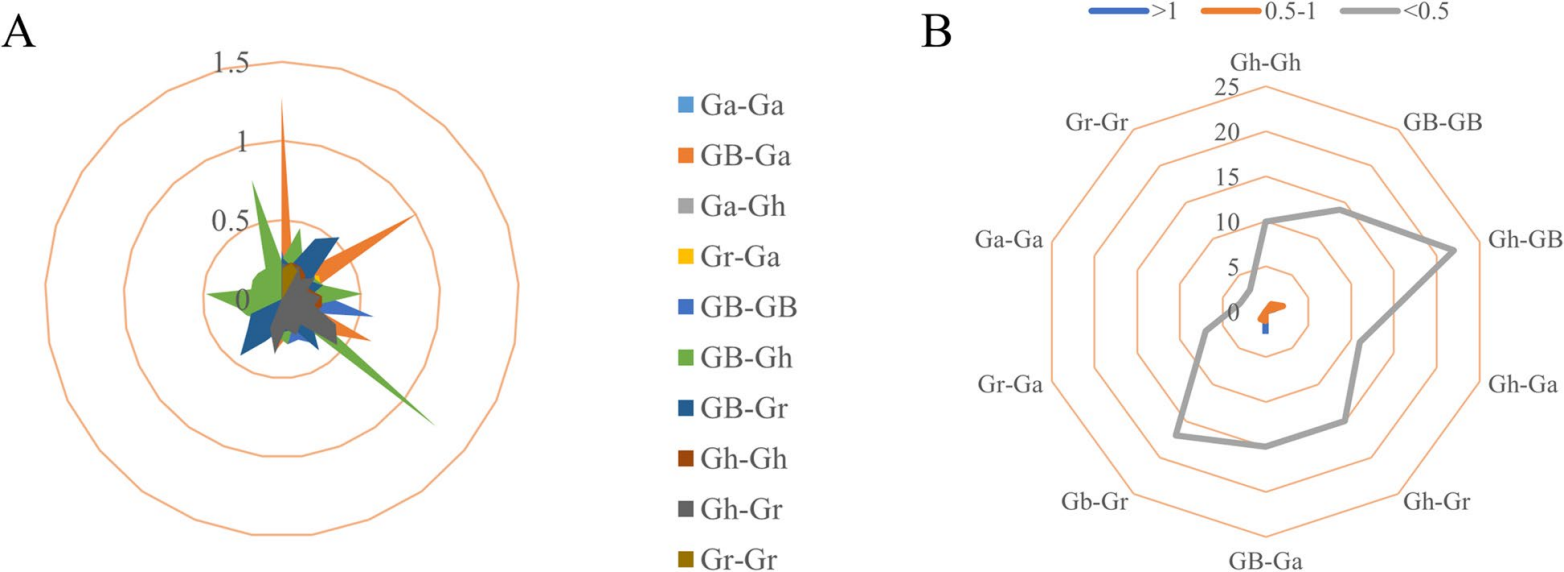
Prediction of the number of duplicated gene pairs involved in different combinations from four *Gossypium* species. **A** Gh represents *G. hirsutum*, GB represents *G. barbadense*, Gr represents *G. raimondii*, Ga represents *G. arboreum*. Different colors represent *Ka / Ks* gene pairs between GB-GB, Gr-Ga, Gr-Gr, Ga-Ga, Gh-GB, Gh-Gh, Gh-Gr, Gh-Ga, GB-Gr, GB-Ga. **B** Displays the number of duplicated gene pairs lies under the extent of selection pressure, legends on top with blue, orange, and grey colors show the range of selection pressure

The *Ka / Ks* ratio can be used as an indicator of whether genes are under positive or negative selection during evolution, The *Ka / Ks* ratios of Gh-Gh, Ga-Gh, Gr-Gh, Gr-Ga, Ga-Ga, GB-GB, GB-Gr and Gr-Gr were all significantly less than 1, 97.6% of which had *Ka / Ks* values less than 1. This indicates that the *AAO* gene formed by the fragment replication event and the fragment replication event mainly underwent purification selection during the evolution process. The replication event may not be seriously differentiated from the source gene, which is relatively conserved. However, in GB-Ga and Gh-GB, there were two and one gene pairs respectively have with *Ka / Ks* values greater than 1, 2.4% of gene pairs had *Ka / Ks* values greater than 1, which also indicated that natural selection changed the protein, rapidly fixed the mutation sites in the population and accelerated gene evolution (Fig. 7). These results indicated that the *AAO* genes have limited functional differentiation after fragment duplication and whole-genome replication. Whether they bring harmful or beneficial traits remains to be studied [25].

### Expression analysis of *GhAAO* genes under different abiotic stresses

**Fig. 8:**
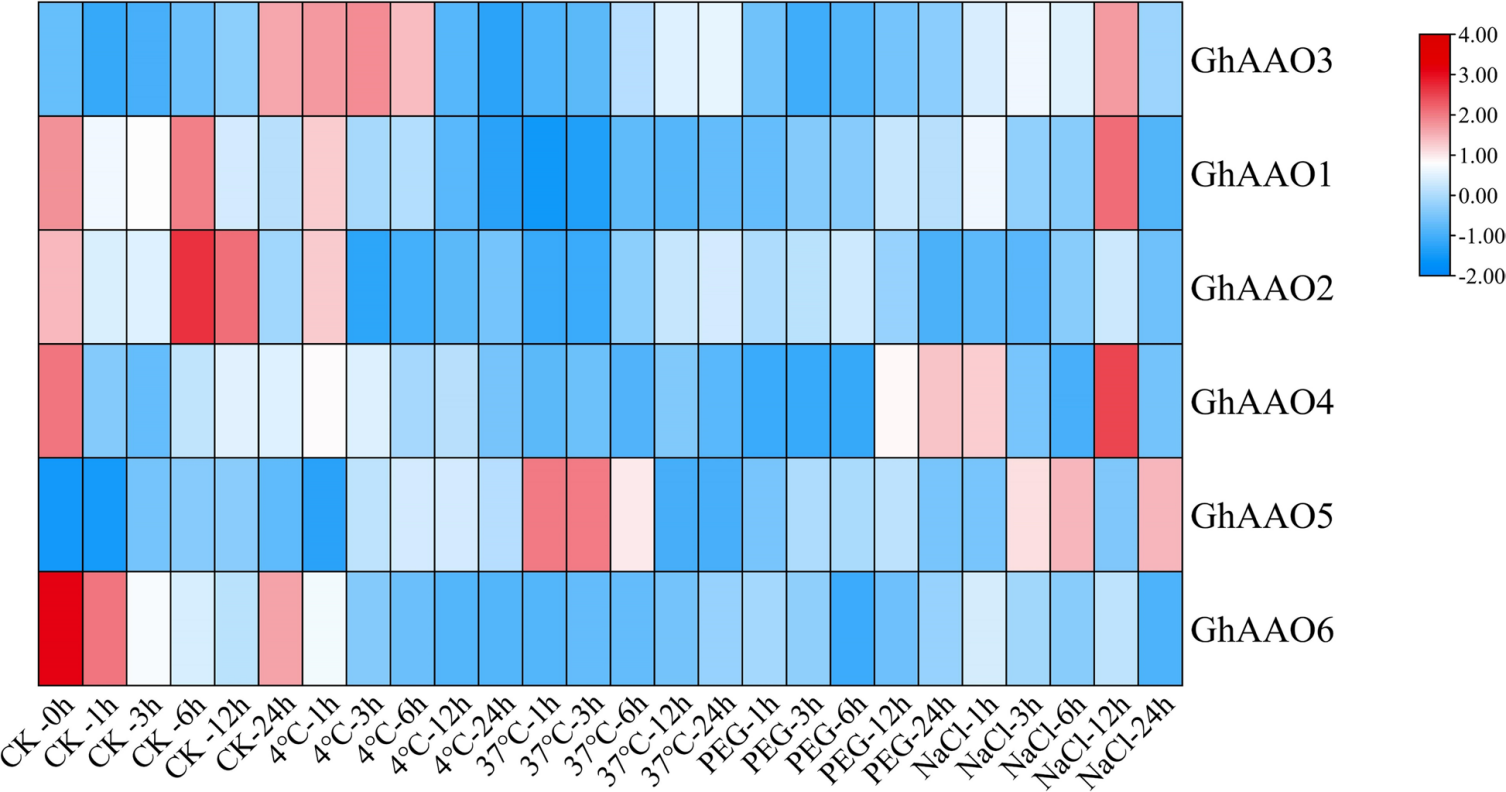
Heatmap of differentially expressed genes (DEGs) of *GhAAO* genes under cold, hot, salt, and PEG stress. The corresponding color of the color band is mapped with the matrix data of the heat map. Colors close to positive values are high expression, and colors close to negative values are low expression. Based on the average gene expression level of the same sample, the expression level higher than the average value is a positive value, and the mark is red. Conversely, expressions below the average value are negative and marked in blue

**Fig. 9:**
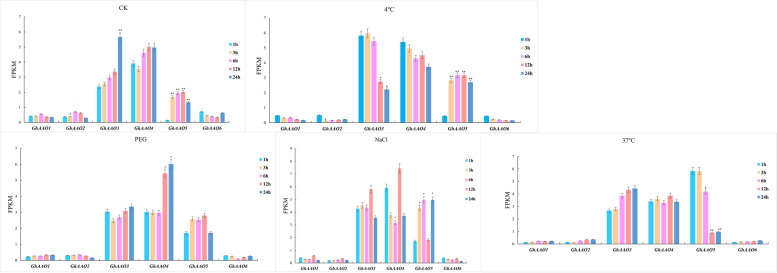
Response of *GhAAO* genes to four abiotic stresses including Salt (250 mM NaCl), Drought (15% PEG6000), Cold (4 ℃) and Heat (37 ℃) at different time periods (1 h, 3 h, 6 h, 12 h and 24 h) shown as Fragments Per Kilobase Million (FPKM)

The RNA-seq data of different stresses was analyzed to show gene expression changes under different stresses (Fig. 8). In addition, we found that *AAO* genes were significantly affected by temperature and salt stress (Fig. 9), *GhAAO5* almost had corresponding response under different stresses, *GhAAO2* was sensitive to low temperature, the expression of *GhAAO4* was significantly increased under drought stress. Overall, the response patterns of *AAO* genes under different stresses were significantly different. We found that the expression of *AAO* genes did not continuously increase or decrease under stress, possibly because plant hormones are trace amounts and they keep dynamic balance in plants [26].

### Expression analysis of different tissues of *AAO* family genes under NaHCO_3_ stress treatment

**Fig. 10:**
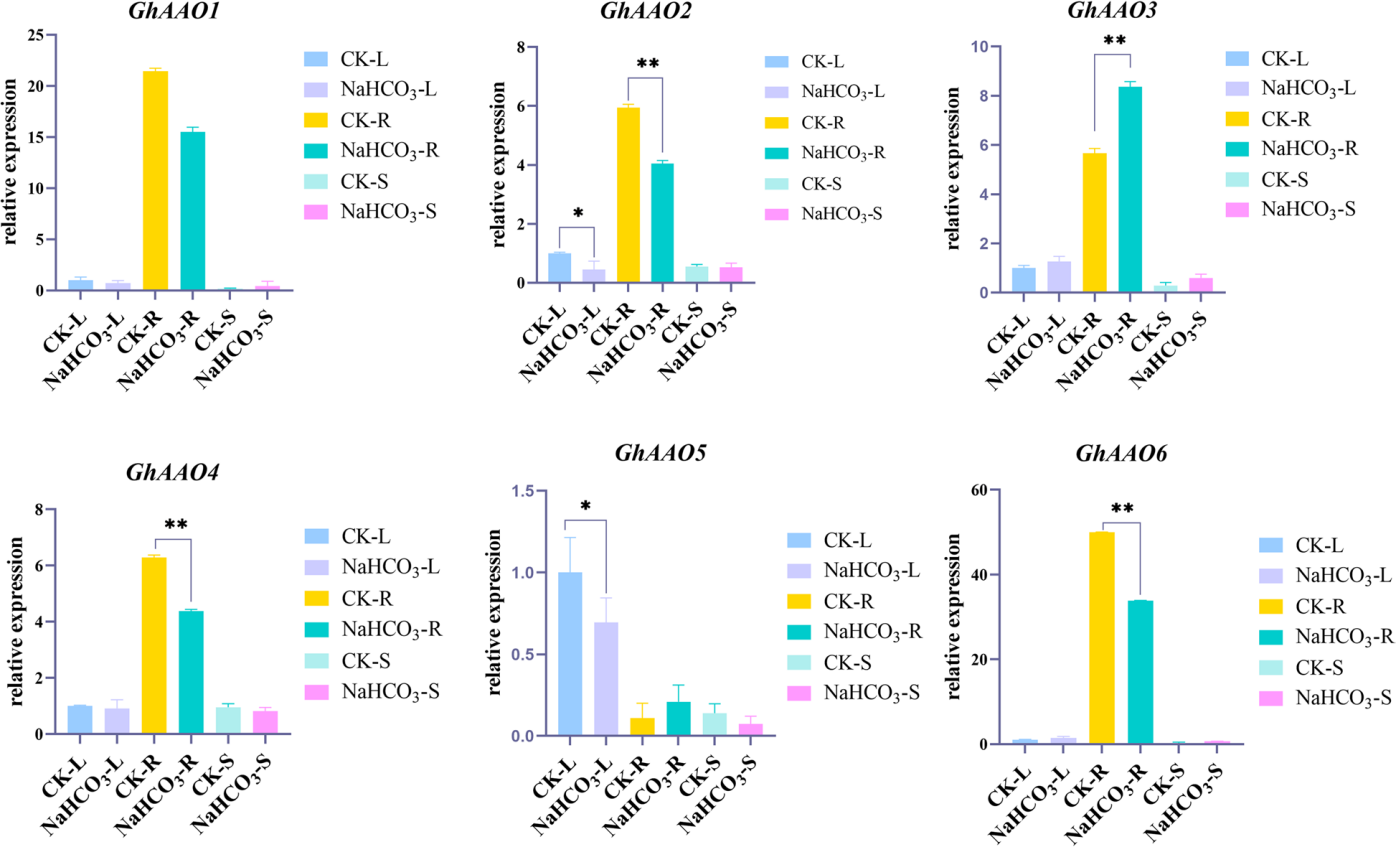
Relative expression of *AAO* genes in different tissues of Zhong9807 under NaHCO_3_ treatment for 12 h. L: leaf; R: root; S: stem. *: 0.01 < *p* < 0.05, **: *p* < 0.01; the resulting mean values were presented as relative units. Error bar represents standard deviation (SD)

The relative expression of *AAO* genes in different tissues under NaHCO_3_ stress was detected by qRT-PCR (Fig. 10). The sequences of primer pairs used are shown in Table S2. We found that the expression of *AAO* family members generally decreased under NaHCO_3_ stress for 12 h, while the expression of *GhAAO3* increased in stems, roots and leaves. Through SPSS analysis, the expression of *AAO* genes in stems showed the most significant difference, so we speculated that cotton might respond to NaHCO_3_ stress by downregulating *AAO* gene expression. Since the differential expression of *GhAAO2* gene in leaves and roots have reached a significant level, we conducted VIGS experiments on the *GhAAO2* gene to verify its function.

### Effect of silencing *GhAAO2* on NaHCO_3_ stress in cotton

**Fig. 11:**
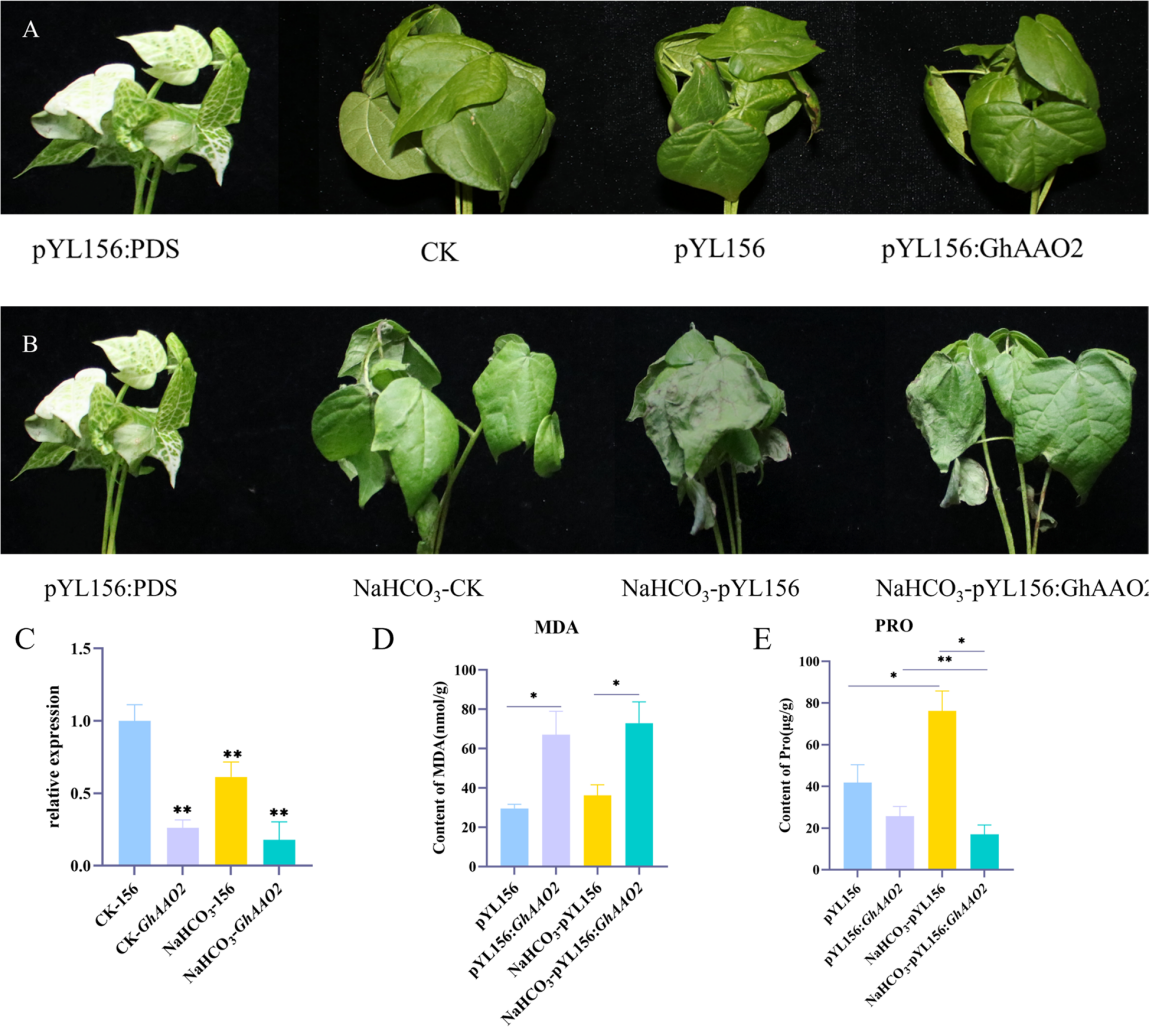
The phenotype of cotton leaves after gene silencing and expression analysis of *GhAAO2* under NaHCO_3_ stress. PDS: positive control. CK: normal plant. pYL156: Blank control with pYL156 empty vector. pYL156: *GhAAO2*: silent plants. NaHCO_3_-CK: Normal plants after NaHCO_3_ treatment. NaHCO_3_-pYL156: Blank control with pYL156 empty vector after NaHCO_3_ treatment. NaHCO_3_-pYL156: *GhAAO2*: silent plants after NaHCO_3_ treatment. A: Phenotype of cotton leaves after gene silencing. B: Phenotype of cotton leaves in silenced plants under NaHCO_3_ stress. C: qRT-PCR for *GhAAO2* under NaHCO_3_ stress. Note: CK-pYL156: Blank control with pYL156 empty vector; CK-*GhAAO2*: silent plants. NaHCO_3_-pYL156: Blank control with pYL156 empty vector after NaHCO_3_ treatment. NaHCO_3_-*GhAAO2*: silent plants after NaHCO_3_ treatment. D: MDA content of empty control and VIGS plants under normal growth and NaHCO_3_ stress. E: PRO content of empty control and VIGS plants under normal growth and NaHCO_3_ stress

After the previous gene expression pattern analysis under NaHCO_3_ stress, we selected *Gh_A09G0970.1* (*GhAAO2*) gene for further functional verification. VIGS vector pYL156: *GhAAO2* was constructed to study the functions of *GhAAO2* under NaHCO_3_ stress. The qRT-PCR results showed that the *GhAAO2* expression level of pYL156: *GhAAO2* was significantly lower than the pYL156, which indicated that VIGS silencing was successful (Fig. 11). When treated with 125 mM NaHCO_3_ for 12 h, we found that the phenotype of the silenced cotton was better than the negative control. The negative control plants had more severe leaves wilting and some veins appeared black (Fig. 11). In the early stage of NaHCO_3_ stress, the expression of *GhAAO2* gene decreased, the cotton responded to NaHCO_3_ stress possibly by adjusting the content of abscisic acid. With the increase in stress time, the damage to plants will be further aggravated. At this point, to resist the stress, the plant may close the pores and wilt the seedling to reduce water and nutrient loss. Abscisic acid can reduce the accumulation of malondialdehyde (MDA), silenced the *GhAAO2* gene, the content of MDA was measured significantly increased. After treated with NaHCO_3_ the content of MDA was increase too, which possibly due to the cotton were injured after NaHCO_3_ treatment. Abscisic acid can promote the accumulation of proline (PRO), After silencing *GhAAO2* gene, the PRO content was significantly reduced. After NaHCO_3_ treatment, the PRO content of the pYL156 control group was increased, the content of PRO has reduced when treated with NaHCO_3_ after *GhAAO2* gene was silenced.

## Discussion

Abiotic stress is one of the main factors to restrict plant growth and development, yield and quality under severe environmental conditions. Salinity and alkaline stresses are the main abiotic stresses factor affecting the plants [27]. As an important cash crop and fiber crop, cotton is widely distributed all over the world. It is a pioneer crop in saline-alkali land but faces severe biotic and abiotic stresses. Abscisic acid aldehyde oxidase is an important enzyme for the synthesis of abscisic acid, but it is rarely reported in cotton.

In this study, 6, 8, 4 and 4 *AAO* genes were identified in four cotton species including *G. hirsutum*, *G. barbadense*, *G. arboreum*, *G. raimondii*. The *AAO* family members of 7 species with close homology were identified by Blastp with e-values of 1e-5 (Blast-2.12.0 +), 4 from *Arabidopsis*, 5 from *Vitis vinifera*, 3 from *Oryza sativa*, 5 from *Zea mays*, 4 from *Glycine max*, 6 from *Populus trichocarpa* and 5 from *Theobroma cacao*. Studies had shown that allotetraploid cotton was formed by inter-genomic hybridization of A-genome diploids and D-genome diploids [28]. In this study, the number of *AAO* family genes in *G. raimondii* and *G. hirsutum* may have lost two genes during evolution.

The results showed that the number of *AAO* genes on the GhAt / GhDt subgenome were different. In the evolutionary analysis, the chromosomal loci of *G. barbadense* and *G. hirsutum* were similar, and the positions of most genes on their chromosomes were highly conserved. The chromosomal loci of *G. barbadense*, *G. arboreum* and *G. hirsutum* were similar, especially the D chromosome loci of tetraploid cotton species still showed a very conservative trend. The diploid cotton *G. raimondii* genes were located at a different locus from the other three cotton species, so we speculated that this may be because *G. raimondii* were wild and had not undergone artificial selection, while the other three cotton species are cultivated and have undergone artificial selection, so there are different loci on the chromosome. Diploid cotton species have strong conservation in the process of evolution to tetraploid cotton species.

Motifs conservative short sequences, which can be used as identification sequence, coding functional protein. Predicting the structure of the *AAO* genes motifs could help us to analysis the family conservative in the process of evolution, provide the basis for understanding gene function and structure of the classification. By structure analyzed we found that four cotton species almost have the same motifs, the lack of individual elements in addition to individual genes, as a whole the evolution of relationship is relatively close [29, 30]. Introns in eukaryotic gene and is excised before translation, which contain a variety of non-coding RNA, also contain some genes transcription regulatory elements, which act as enhancers or silencers [31].

The *cis*-elements related to the stress were predicted in *AAO* genes, the *cis*-elements can provide the site of action, then *cis*-elements interactions with *trans*-acting factors, regulation of gene expression. Although the *AAO* genes contains *cis*-elements each are not identical, but all of them are included with the stress response components. Combined with the structure of the gene sequences, we found that some correlation between redundant structures and *cis*-element. The *AAO* genes contain a large number of plant hormones elements, five major plant hormones were covered, therefore, we hypothesized that inducing plant hormones related transcription factors and corresponding *cis*-element function, regulate the expression of *AAO* family genetic changes to enable plants to respond to abiotic stress [32].

Studies showed that the plants to adapt to environmental change, evolution occurs in the form of whole genome duplications, fragment duplications or tandem duplications. Dicotyledonous plants through whole genome duplication event about 130 million years ago, and cotton has experienced the whole genome duplication event in 60 million years ago, *G. raimondii* in evolution experienced at least two whole genome replications [33]. The A / D genome of diploid cotton was separated 5–10 Myr years ago [34]. *G. hirsutum* was generated from the hybridization of *G. raimondii* and *G. arboreum*, the number of chromosomes was doubled 1–2 Myr years ago [35]. However, *AAO* family members are not uniformly distributed across chromosomes, according to the calculation of selection pressure, most *AAO* genes had undergone purification selection and were relatively conserved.

When plants are subjected to abiotic stress, many signal transduction pathways are activated, a series of adaptation mechanisms are stimulated [36]. The plants respond to stress through changes in cell and physiology [37]. QRT-PCR results of *AAO* genes in different tissues under NaHCO_3_ stress show that *AAO* gene family responds to NaHCO_3_ stress by down-regulating the expression genes. Moreover, we found that significant differential expression of *GhAAO2* occurred in leaves and roots. In addition, we also silenced *GhAAO2* gene by VIGS, and the silenced *GhAAO2* plants showed better tolerance under NaHCO_3_ stress compared with the negative control plants, which also confirmed that this gene was indeed a negative regulatory gene under NaHCO_3_ stress.

Aldehyde oxidase gene belongs to a multi-gene family, and the number of *AAO* genes varies in different species. Through the study of the evolutionary relationship of *AAO* genes, it is believed that the occurrence of gene duplication and gene repression events have led to the differences in plants, vertebrates and insects. The generation of aldehyde oxidase isoforms is generally considered to be the ancestor of all *AAO* genes. *AAO* genes originally evolved from the *XDH* genes through at least two independent gene duplication events. The physiological function of aldehyde oxidase was first studied in plants. Plant *AAO* genes are thought to play a catalytic role in the synthesis of abscisic acid and indoleacetic acid, thereby participating in many aspects of plant growth, development, and environmental adaptation. The researchers found different numbers of aldehyde oxidase genes in different plant species separately and performed an evolutionary analysis of them, finding that the genes were clustered together. However, amino acid residue similarity between *AAO* genes within species is higher than between species. This suggests that it is possible that all plant aldehyde oxidase genes originated from the same ancestor and evolved independently in the form of gene duplication [38]. In plants, it participates in the synthesis of abscisic acid and indoleacetic acid, which in turn regulates plant growth, development and adaptation to the environment [39]. In mammals, aldehyde oxidase studies have focused on drug and exogenous metabolism [40].

In plants, guard cells can sense environment information from the leaves, including abiotic stresses and biotic stresses, abscisic acid signaling plays a vital role in the face of these stresses. Ion channels are key signaling elements mediated by abscisic acid in stomatal movement. In guard cells, abscisic acid can regulate cell ion flux, mediate stomatal closure, reduce water loss, maintain physiological and metabolic balance in plants under stress conditions. *AAO* can regulate the synthesis of ABA in roots and leaves, thereby alleviating the damage to plants. Osmotic stress can cause the expression of *AAO* [41]. ABA synthesis may be regulated by *NCED* and *AAO* in drought [42], ABA plays an important role in salt stress signal transduction, under salt stress, the activity of *AAO* in roots and leaves of *Pisum sativum* was enhanced [43]. Relevant reports have found that the level of *AAO* mRNA in roots of *Arabidopsis thaliana* under salt stress is also significantly increased [44]. *AAO* activity was detected in the roots of *Lolium perenne* and *Hordeum vulgare* under salt and ammonium treatment, the change was more obvious in the roots than in the leaves [45].

**Fig. 12:**
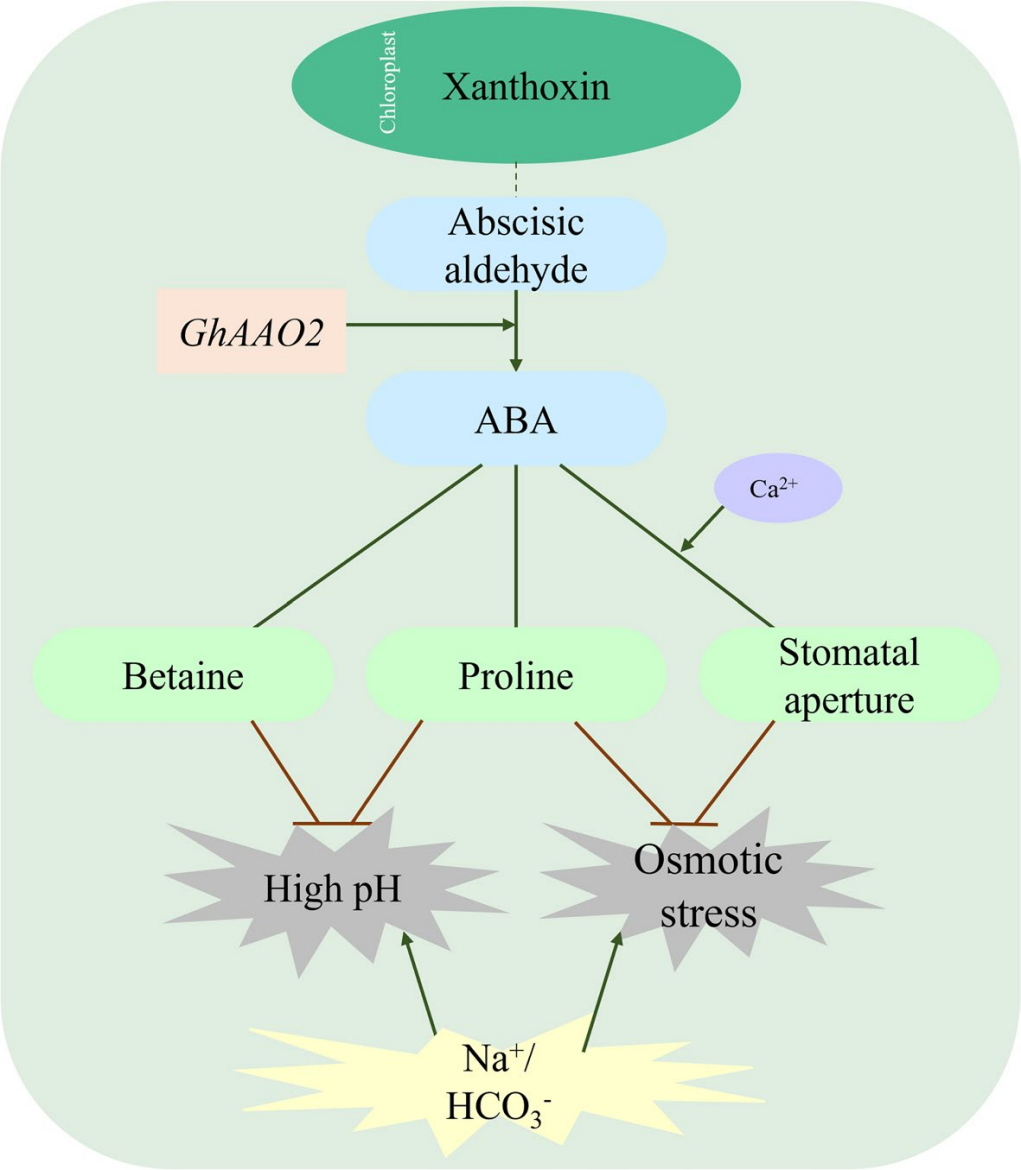
Mechanism of *GhAAO2* in regulating cotton response to NaHCO_3_ stress

According to the expression of *AAO* genes in different tissues under NaHCO_3_ stress, the expression pattern of *AAO* genes under different abiotic stress, and the types of *cis*-elements contained, *GhAAO2* was selected for further study. The wilting degree of seedlings after silencing *GhAAO2* gene was found to be less severe than that of the negative control. We speculated that *GhAAO2* might play an important role in responding to NaHCO_3_ stress (Fig. 12). The change of ion concentration in plant root environment under NaHCO_3_ stress, large accumulation of ions could reduce soil water potential, which made plants suffer osmotic stress [46], root cells could not take advantage of the water potential difference to absorb water from the soil would cause physiological drought of plants, which affect the growth and development of plants. It was found that abscisic acid could regulate the synthesis of osmotic regulatory substances proline and betaine, relieved the damage from high pH, maintained the stability of cell membrane structure, Abscisic acid also activates Ca^2+^ channels in guard cells. Regulating the movement of ions in and out of cells alters the turgor pressure surrounding cells, thus inhibiting stomatal open or close [47].

## Conclusion

This study comprehensive analyzed the structure and chromosome distribution of the *AAO* genes, and found the *AAO* genes keep conservative in the process of evolution, *cis*-element analysis that found a lot of stress response elements, we analyzed the different tissues response NaHCO_3_ stress and *AAO* genes response of different stresses. We selected the *GhAAO2* gene for further function verification, which confirmed that this gene could regulates NaHCO_3_ stress. These results enrich our understanding of cotton *AAO* genes and provide reference for the study of candidate genes related to stress response.

## Material and methods

### Databases

To identify the members of the *GhAAO* gene family, the protein sequence and genome annotation files of four cotton *G. arboreum* (CRI); *G. raimondii* (JGI); *G. hirsutum* (NAU) and *G. barbadense* (ZJU) were downloaded from Cotton Functional Genomic Database (CottonFGD) (https://cottonfgd.org/) [48]. Genome data of other seven species: *Arabidopsis thaliana*, *Vitis vinifera*, *Populus trichocarpa*, *Theobroma cacao*, *Glycine max*, *Oryza sativa,* and *Zea mays* were retrieved BLAST (Basic Local Alignment Search Tool) was downloaded from NCBI (https://www.ncbi.nlm.nih.gov/) [49].

### Identification of *AAO* family members

The Hidden Markov Model (HMM) profile of PF02738 was downloaded from the Pfam (https://pfam.xfam.org/) website, all the possible members of *AAO* gene family were retrieved by using hmmer (version 3.3.1) (http://www.hmmer.org/) [50] and BLAST (Basic Local Alignment Search Tool). Using NCBI Batch Web CD-Search Tool (https://www.ncbi.nlm.nih.gov/Structure/cdd/wrpsb.cgi) to screen the genes with default parameters which most probably belongs to *AAO* gene family. We renamed the identified *AAO* family members *GhAAO1*-*GhAAO6* (Gh stands for *G. hirsutum*, *AAO* stands for abscisic acid aldehyde oxidase, and 1–6 are serial numbers).

### Phylogenetic analysis and multiple sequences alignments

In order to study the evolutionary relationship between *AAO* genes in four cotton species, the *AAO* genes were provided in MEGA software (version 7.0) [51], analyzing the multiple sequence alignment by using ClustalW algorithm. Subsequently, the phylogenetic tree was constructed by using the Maximum Likelihood (ML) method in MEGA software [52]. The Blastp soft was used to obtain homologous genes of other species (*Theobroma cacao* (Tc), *Arabidopsis thaliana* (At), *Oryza sativa* (Os), *Populus trichocarpa* (Pt), *Vitis vinifera* (Vv), *Glycine max* (Gm), *Zea mays* (Zm)) with the parameters evalue 1e-5. Then we used MEGA7.0 software to multiple sequences alignments [53], constructed the phylogenetic tree with Maximum Likelihood (ML) method [54]. Online website EvolView (https://evolgenius.info//evolview-v2/#login) was used to beautify the obtained phylogenetic tree.

### Chromosomal distribution of *AAO* genes from four *Gossypium* species

To study the chromosomal distribution of *AAO* gene family members in four cotton species, physical positions of chromosomal locations from four cotton species including *G. hirsutum*, *G. arboreum*, *G. raimondii*, and *G. barbadense* were visualized by TBtools software [55]. Genome annotation file, coding sequences of four cotton species were downloaded from CottonFGD [48].

### Analysis of the conserved protein motifs and gene structure

Multiple Em for Motif Elicitation (https://meme-suite.org/meme/) was applied to analyze *AAO* genes conserved motifs [56]. MAST file predicted in MEME website, phylogenetic tree analysis of NWK file, GFF3 genome annotation file of *Gossypium* were put into these files in TBtools software to visualized the evolutionary relationship, gene structure, and conserved motifs of *GhAAO* genes.

### Analysis of *AAO* gene family members’ promoter regions

The promoter sequences 2 kb upstream of the start codon of *GhAAO* genes were downloaded from the CottonFGD, using PlantCARE (http://bioinformatics.psb.ugent.be/webtools/plantcare/html/) to extract *cis*-acting elements related to stress response in the promoter region of *GhAAO* genes.

### Collinearity analysis of *AAO* genes in four *Gossypium* species

To investigate the collinearity and analyze the syntenic relationship among *AAO* genes of four cotton species, the collinear and homologous chromosomal regions among four cotton species visualized in the circos in TBtools [55]. Diagram was illustrated by using the genome annotation file and complete genome sequences of these cotton species by MCScanX tool [57].

### Calculation of selection pressure

CDS sequences of the *AAO* genes in the four cotton species were downloaded from CottonFGD. 125 pairs of gene pairs obtained from 10 combinations of collinear were used to calculate the ratio of the number of nonsynonymous substitutions to the number of synonymous substitutions, the MEGAX comparison method was used to identify the selection pressure to analyze the selection pressure in the evolution of the genes [28].

### Differentially expressed genes under different abiotic Stresses

The RNA-Seq data (PRJNA490626) of cotton under salt, PEG, cold, and heat stress was downloaded from the online website (http://grand.cricaas.com.cn/page/tools/expressionVisualization), the heat map along with phylogenetic tree was generated through TBtools software to analyze these genes in the expression level under different stresses [58].

### Expression analysis of different tissues of *AAO* family genes under NaHCO_3_ stress treatment

According to the previous exploration of the conditions of NaHCO_3_ stressed cotton in our laboratory, we finally chose 125 mM NaHCO_3_ to treat cotton at the three leaves and one heart stage for 12 h (water treatment was the control). The roots, stems and leaves were taken, and the RNA was extracted, then reverse transcribed into cDNA for fluorescence quantitative experiment [59]. The *AAO* genes of different tissues were analyzed under the NaHCO_3_ stress.

### VIGS and alkaline treatment

The 300 bp silencing fragment was selected from the online website (https://vigs.solgenomics.net/), the purified fragment was inserted into the pYL156 vector fragment. The Agrobacterium carrying pYL156 (Blank control, empty vector), pYL156-*GhAAO2* and PDS (Positive control) were mixed with pYL192 (Helper Bacteria) were injected in cotyledons of Zhong 9807. After 24 h in the dark, then cultured them normally, when the leaves turned white in the Positive control, indicating that the experiment was successful. When cotton grew to the three leaves and one heart phase, treated with 125 mM NaHCO_3_. Phenotypes were observed and samples were taken for quantitative fluorescence experiments to further determine whether genes were silenced.

## Supplementary Information

**Additional file 1.**

**Additional file 2 .**

## Data Availability

The datasets generated and/or analyzed during the current study are available in the CottonFGD (https://cottonfgd.org/), RNA-Seq data downloaded from NCBI (https://www.ncbi.nlm.nih.gov/) under accession number (PRJNA490626).

## Abbreviations

ABA: Abscisic acid
AAO: Abscisic acid aldehyde oxidase
SA: Salicylic acid
ET: Ethylene
MeJA: Methyl jasmonate
FPP: Farnesyl pyrophosphate
HMM: Hidden Markov Model
VIGS: Virus-induced Gene Silencing
Ga: *Gossypium arboreum*
Gh: *Gossypium hirsutum*
Gr: *Gossypium raimondii*
Gb: *Gossypium barbadense*
ZEP: Zeaxanthin epoxidase
NCED: 9-c*is*-epoxycarotenoid dioxygenase
SDR: Short-chain dehydrogenase
FAD: Flavin adenine dinucleotide
XDH: Xanthine dehydrogenase
At: *Arabidopsis thaliana*
Vv: *Vitis vinifera*
Pt: *Populus trichocarpa*
Tc: *Theobroma cacao*
Gm: *Glycine max*
Os: *Oryza sativa*
Zm: *Zea mays*
qRT-PCR: Quantitative real-time polymerase chain reaction
FPKM: Fragments Per Kilobase Million
MDA: Malondialdehyde
PRO: Proline
